# Low risk of thromboembolic complications after fast-track hip and knee arthroplasty

**DOI:** 10.3109/17453674.2010.525196

**Published:** 2010-10-08

**Authors:** Henrik Husted, Kristian Stahl Otte, Billy B Kristensen, Thue Ørsnes, Christian Wong, Henrik Kehlet

**Affiliations:** ^1^Department of Orthopedic Surgery, Hvidovre University Hospital; ^2^Department of Anesthesiology, Hvidovre University Hospital; ^3^Section of Surgical Pathophysiology, Rigshospitalet, Copenhagen University; ^4^the Lundbeck Centre for Fast-track Hip and Knee Arthroplasty, Copenhagen, Denmark

## Abstract

**Background and purpose:**

Pharmacological prophylaxis can reduce the risk of deep venous thrombosis (DVT), pulmonary embolism (PE), and death, and it is recommended 10–35 days after total hip arthroplasty (THA) and at least 10 days after total knee arthroplasty (TKA). However, early mobilization might also reduce the risk of DVT and thereby the need for prolonged prophylaxis, but this has not been considered in the previous literature. Here we report our results with short-duration pharmacological prophylaxis combined with early mobilization and reduced hospitalization.

**Patients and methods:**

1,977 consecutive, unselected patients were operated with primary THA, TKA, or bilateral simultaneous TKA (BSTKA) in a well-described standardized fast-track set-up from 2004–2008. Patients received DVT prophylaxis with low-molecular-weight heparin starting 6–8 h after surgery until discharge. All re-admissions and deaths within 30 and 90 days were analyzed using the national health register, concentrating especially on clinical DVT (confirmed by ultrasound and elevated D-dimer), PE, or sudden death. Numbers were correlated to days of prophylaxis (LOS).

**Results:**

The mean LOS decreased from 7.3 days in 2004 to 3.1 days in 2008. 3 deaths (0.15%) were associated with clotting episodes and overall, 11 clinical DVTs (0.56%) and 6 PEs (0.30%) were found. The vast majority of events took place within 30 days; only 1 death and 2 DVTs occurred between 30 and 90 days. During the last 2 years (854 patients), when patients were mobilized within 4 h postoperatively and the duration of DVT prophylaxis was shortest (1–4 days), the mortality was 0% (95% CI: 0–0.5). Incident cases of DVT in TKA was 0.60% (CI: 0.2–2.2), in THA it was 0.51% (CI: 0.1–1.8), and in BSTKA it was 0% (CI: 0–2.9). Incident cases of PE in TKA was 0.30% (CI: 0.1–1.7), in THA it was 0% (CI: 0–1.0), and in BSTKA it was 0% (CI: 0–2.9).

**Interpretation:**

The risk of clinical DVT, and of fatal and non-fatal PE after THA and TKA following a fast-track set-up with early mobilization, short hospitalization, and short duration of DVT prophylaxis compares favorably with published regimens with extended prophylaxis (up to 36 days) and hospitalization up to 11 days. This calls for a reconsideration of optimal duration of chemical thromboprophylaxis.

Total hip and knee arthroplasty (THA and TKA) are associated with perioperative risks including deep venous thrombosis (DVT) and pulmonary embolism (PE)—both of which are manifestations of venous thromboembolism (VTE)—possibly leading to a post-thrombotic syndrome (PTS) or death. The mechanisms underlying VTE are not fully understood (Malone 1977, Malone and Agutter 2006). Immobility with long hospitalization after surgery may be a contributory factor (Heit et al. 2001, Seddighzadeh et al. 2007, Sharma et al. 2007).

The recent evidence-based guidelines for DVT prophylaxis according to the American College of Chest Physicians consist of at least 10 days of prophylaxis after TKA and THA, and preferably up to 35 days after THA (Geerts et al. 2008). The incidence of DVT has been found to have decreased over time, possibly as a result of increased and earlier mobilization (Xing et al. 2008). We therefore present our data on DVT, PE, and death after TKA, bilateral simultaneous TKA (BSTKA), and THA with a fast-track set-up with short DVT prophylaxis, early mobilization, and short hospitalization.

## Patients and methods

The fast-track set-up was implemented in 2003 and consists of optimized logistics and evidence-based clinical features (as described in detail below) (Husted et al. 2008). Optimization of logistics includes a homogenous ward with arthroplasty patients only, regular staff, high continuity, preoperative information including intended length of stay (LOS), admission on the day of surgery, and functional discharge criteria (ability to get dressed, to get in and out of bed, to get into and up from a chair, and to walk with crutches or better).

Since then, all patients operated on with THA, TKA, bilateral simultaneous TKA (BSTKA), and revisions have been enrolled in the program. We concentrated on pain treatment and early mobilization, and our current research involves refinement of these tools for improving convalescence in order to reduce length of stay. Thus, LOS has been reduced in increments. From 2004 to 2006 the intended LOS was around 5 days, and from 2007 to 2008 the intended LOS was 3 days when local infiltration analgesia (LIA) was introduced, allowing patients to be mobilized with only moderate pain (Andersen et al. 2008a, b, c, Kerr and Kohan 2008).

The patients were consecutive and unselected, and all of them were operated using regional anesthesia—from 2004 to 2006 epidural for TKAs and spinal for THAs, but from 2007 all with spinal anesthesia with 2 mL hyperbaric bupivacaine 0.5% (TKA), 2.5 mL plain bupivacaine 0.5% (THA), and 3 mL plain bupivacaine 0.5% (BSTKA) combined with local infiltration analgesia (LIA) (Andersen et al. 2008a). Also, a standardized program in the operating theater was followed, including fluid administration (Holte et al. 2007), use of tranexamic acid (Benoni and Fredin 1996, Husted et al. 2003), small standard incisions (posterior for THA, midline skin and medial parapatellar for TKA), no drains, use of compression bandages (Andersen et al. 2008c), and cooling (Kullenberg et al. 2006). Multimodal opioid-sparing analgesia (NSAID, paracetamol, gabapentin, local analgesia) together with early mobilization has facilitated the reduction in LOS (Andersen et al. 2009).

All TKA patients were operated with a tricompartmental cemented prosthesis. Surgery was done in a bloodless field by using a femoral tourniquet (inflated to 100 mmHg above systolic blood pressure) from incision until cementation of the prosthesis was finished. 15 minutes before incision, 500 mg of intravenous tranexamic acid was administered and another 500 mg just before tourniquet release.

All THA patients were operated with an uncemented cup and either a cemented or an uncemented stem. 15 minutes before incision, 1,000 mg of intravenous tranexamic acid was administered. Immediate and full weight bearing was allowed after all operations.

Discharge criteria were strictly functional and all patients were discharged to their own homes. LOS was counted as number of nights of hospitalization after the operation.

DVT prophylaxis consisted of low-molecular-weight heparin (LMWH) (enoxaparin 40 mg subcutaneously) starting 6–8 h postoperatively and continuing once daily in the evening until discharge. No extended prophylaxis was given and no mechanical devices (including compression stockings) were used. No attempt was made to identify high-risk patients; all patients received the same regime.

Patients were mobilized on the day of operation. In the period 2004–2006, patients were mobilized in the afternoon or evening, but from 2007 the LIA technique made a more aggressive mobilization possible, usually within 2–4 h after the operation. The pain treatment consisted initially of NSAIDs and paracetamol—with opioids for breakthrough pain—but since 2007, a standardized pain treatment has been given to all patients (gabapentin, paracetamol, celecoxib with oxycodone 10 mg on request) (Andersen et al. 2008a, b).

All patients are registered in the National Health Register when they are operated and all re-admissions at any hospital in Denmark are registered. All records were scrutinized for re-admissions, and all re-admissions with DVT, PE, or death and also deaths without re-admission were analyzed and registered. All deaths were followed by autopsy except in one case, and the diagnosis was recorded and linked to the operation if a clotting episode was involved. In the case where autopsy was not performed, death was assumed to be linked to the operation. No patients were lost to follow-up, which was therefore 100%.

### Statistics

The study was presented to the ethical committee for the capital region. Numbers of incident cases are the cumulated cases, i.e. the number of new cases within a specified time period divided by the size of the population initially at risk. 95% confidence intervals (CI) were calculated using the Wilson procedure without correction of continuity using the VassarStats website for statistical computation (faculty.vassar.edu/lowry/VassarStats.html). All data analyses were conducted with SPSS for Windows, version 13.0.

## Results

From 2004 through 2008, 1,977 operations were performed: 947 THA, 784 TKA, and 246 BSTKA. Mean length of stay (LOS) decreased during the study period from 4.6 to 3.1 days for TKA, 6.3 to 3.9 days for THA, and 7.3 to 4.2 days for BSTKA.

Tables 1 and 2 show distribution of operations, LOS, and corresponding numbers of incident cases of DVT, PE, and death within 90 days. The figure shows the numbers of incident cases of DVT, PE, and death for each year with corresponding mean LOS.

**Table 1.** Distribution of operations and length of stay (LOS)

	No. of operations				Length of stay		
	All	TKA	BSTKA	THA	TKA	BSTKA	THA
2004	311	124	28	159	4.4	5.9	6.3
2005	378	167	32	179	4.6	6.0	5.3
2006	434	159	58	217	4.5	7.3	5.7
2007	433	182	52	199	3.1	4.2	4.2
2008	421	152	76	193	3.2	4.2	3.9
Total	1,977	784	246	947			

**Table 2.** Numbers and incidences (with 95% CI) of deep venous thrombosis (DVT), pulmonary embolism (PE), and death (D) within 90 days

	Number of				Incidences (CI)	
	DVT	PE	D	DVT	PE	D
TKA						
2004	2	0	1	1.61 (0.4–5.7)	0 (0–3.0)	0.81 (0.1–4.4)
2005	4	2	1	2.40 (0.9–6.0)	1.20 (0.3–4.3)	0.60 (0.1–3.3)
2006	1	1	0	0.63 (0.1–3.5)	0.63 (0.1–3.5)	0 (0–2.4)
2007	1	1	0	0.55 (0.1–3.1)	0.55 (0.1–3.1)	0 (0–2.1)
2008	1	0	0	0.66 (0.1–3.6)	0 (0–2.5)	0 (0–2.5)
Total				1.15 (0.6–2.2)	0.51 (0.2–1.3)	0.26 (0.1–1.0)
BSTKA						
2004	0	0	0	0 (0–12.1)	0 (0–12.1)	0 (0–12.1)
2005	0	0	0	0 (0–10.7)	0 (0–10.7)	0 (0–10.7)
2006	0	0	0	0 (0–6.2)	0 (0–6.2)	0 (0–6.2)
2007	0	0	0	0 (0–6.9)	0 (0–6.9)	0 (0–6.9)
2008	0	0	0	0 (0–4.8)	0 (0–4.8)	0 (0–4.8)
Total				0 (0–1.5)	0 (0–1.5)	0 (0–1.5)
THA						
2004	0	1	1	0 (0–2.4)	0.63 (0.1–3.5)	0.63 (0.1–3.5)
2005	0	1	0	0 (0–2.1)	0.56 (0.1–3.1)	0 (0–2.1)
2006	0	0	0	0 (0–1.7)	0 (0–1.7)	0 (0–1.7)
2007	1	0	0	0.50 (0.1–2.8)	0 (0–1.9)	0 (0–1.9)
2008	1	0	0	0.52 (0.1–2.9)	0 (0.1–2.0)	0 (0.1–2.0)
Total				0.21 (0.1–0.8)	0.21 (0.1–0.8)	0.11 (0–0.6)

### Deaths

From 2004 through 2008, 14 patients (0.71% (CI: 0.4–1.2)) died within 90 days of surgery: 3 in 2004 (mesenterial thrombosis (89 days), myocardial infarction (2 days), and sepsis/pneumonia (27 days)); 3 in 2005 (hepatic cirrhosis and coma (28 days), perforated colon cancer (51 days), sudden death (1 day, no autopsy)); 3 in 2006 (metastatic lung cancer (77 days), dysregulated warfarin treatment (resulting in massive bleeding) for atrial fluttering (50 days), perforated gastric ulcer (22 days)); 4 in 2007 (cardiomyopathy and failure (89 days), lung cancer (10 days), mechanical bowel obstruction (76 days), hepatic cirrhosis with esophageal bleeding (83 days)); and 1 in 2008 (aortic stenosis, pulmonary insufficiency, and cardiac failure (19 days)). Thus, 2 deaths in 2004 (1 THA and 1 TKA) and 1 in 2005 (TKA)—and none in 2006–2008—may have been related to clotting episodes, giving an overall risk of VTE-related mortality of 0.15% (CI: 0.04–0.48).

### Pulmonary embolism

From 2004 through 2008, 6 patients had confirmed pulmonal embolism (PE) diagnosed by perfusion-ventilation lung scintigraphy, with none in the BSTKA group, 4 in the TKA group, and 2 in the THA group leading to risks from 0% to 0.63%. All PE occurred before 30 days postoperatively. No patients died from PE (except the potential one with sudden unexplained death without autopsy in 2005). During the last 2 years with the shortest LOS, the risk of PE was 0.30% (CI: 0.1–1.7) after TKA and 0% (CI: 0–1.0) after THA.

**Figure:**
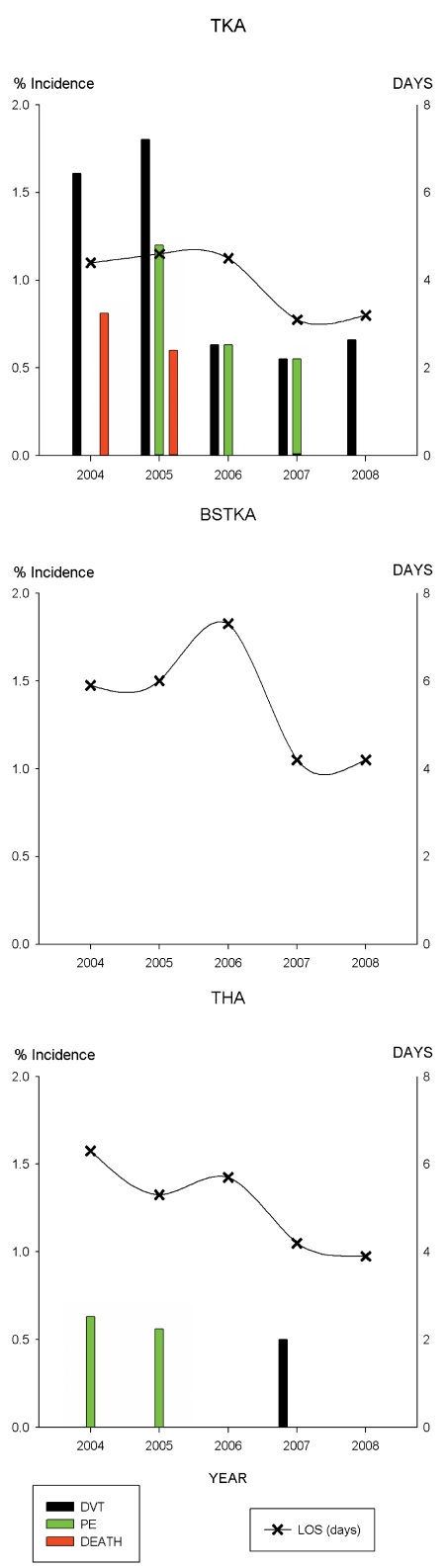
Numbers of incident cases of DVT, PE and death for each year with corresponding mean LOS.

### Deep venous thrombosis

11 clinical cases of DVT were found within 90 days from 2004 through 2008 with the clinical diagnosis being confirmed by an elevated D-dimer result and a positive ultrasound examination: 9 in the TKA group (1.2%) and 2 in the THA group (0.2%). No DVT was seen in the BSTKA group. Only 2 cases of DVT were found between 30 and 90 days (1 THA, 1 TKA). None of the patients with DVT developed PE within 90 days, since all patients with PE had no clinical diagnosis of DVT. 1 of 2 DVTs in THA was proximal to the knee joint, and 3 of 9 DVTs in TKA. Combining the last 2 years with the shortest LOS, the risk of DVT in TKA was 0.6% (CI: 0.2–2.2) and 0.51% (CI: 0.1–2.1) after THA.

97 days of hospitalization for DVT/PE re-admissions were used in the TKA group (21 days, 55 days, 12 days, 8 days, and 1 day for the years 2004–2008, respectively), 0 days for the BSTKA group, and 28 days for the THA group (12 days, 4 days, 0 days, 10 days, and 2 days for 2004–2008, respectively). In addition, 80 patients were re-admitted for suspected DVT during the study period (46 TKAs, 1 BSTKA, and 33 THAs), but the diagnosis was excluded by ultrasound and D-dimer measurements.

## Discussion

We found a very low risk of clinically symptomatic VTE and deaths potentially related to the operation in an unselected patient population with a consecutive set-up and 100% follow-up for 90 days. We used the unique Danish National Health Register to record all national re-admissions and the Danish Personal Register to assess mortality. In Denmark, all patients with verified DVT are re-admitted and treated in hospital, so we believe that the numbers of incident cases we found are true risks and not underestimates of the real numbers—as no patients are treated initially for DVT in an outpatient setting. Also, all charts were scrutinized for all types of re-admission in order to avoid missing cases of VTE that were miscoded as other diagnoses for re-admission.

We found an overall risk of death potentially related to the operation of 0.15%, which compares favorably with the results of a recent study where 0.31% of patients died within 90 days of an operation with THA or TKA, and following 6 weeks of 150 mg aspirine daily as the only prophylaxis (Cusick and Beverland 2009). Also, the risk of death possibly resulting from clotting episodes found in the current study was lower or similar to those in all previously published studies where postoperative mortality was validated, regardless of type (whether chemoprophylactic or mechanical) and duration of thromboprophylaxis (up to 36 days) (Mantilla et al. 2002, Blom et al. 2006, Tarity et al. 2006, Lachiewiicz and Soileau 2006, 2007, Samama et al. 2007, Eriksson et al. 2008, Aynardi et al. 2009, Lassen et al. 2009).

Rebound activation of coagulation after cessation of prophylaxis may play a role in VTE and death after surgery. Rebound hypercoagulability has been described to occur after discontinuation of chemical prophylaxis (Kontny 1997, Cundiff 2008) based on continuous generation of platelets and thrombin after cessation of the prophylactic drug. Rebound hypercoagulability is estimated to account for 2% of patients having VTE in the first 2 months after discontinuing oral anticoagulants; it is also been found, however, that an increased duration of treatment with oral anticoagulants does not reduce the overall number of adverse events (Cundiff 2008). However, the low risk of VTE in our study suggests that rebound hypercoagulability may not be a problem after a few days of thromboprophylaxis.

The evidence-based guidelines for DVT prophylaxis according to the American College of Chest Physicians involve at least 10 days of treatment after TKA and THA—and preferably up to 35 days after THA (Geerts et al. 2008). LMWH is recommended, and has been found to reduce the risk of VTE by 50%, whereas these guidelines do not support the sole use of aspirin as a prophylactic agent.

The antiplatelet effect of aspirin is, nevertheless, the reason for the use of this drug in many hospitals, in the USA especially but also in Europe. Cusick and Beverland (2009) advocated prophylaxis with aspirin (150 mg daily for 6 weeks) only for the standard patient, except for patients with a prior history of PE. They concluded that: “…with modern surgical practice, elective hip and knee replacement should no longer be considered high-risk procedures.” Others have supported this belief. Thus, some propose differentiated DVT prophylaxis by identifying low- and high-risk patients after THA and TKA, balancing the need for prophylaxis against the risk of bleeding (Lachiewicz and Soileau 2006, 2007, Dorr et al. 2007). Such regimens have led to incidences of 0.25% for (non-fatal) PE after THA and TKA (Dorr et al. 2007), and 0.35% for PE after TKA and 0.7% after THA (Lachiewicz and Soileau 2006 and 2007). These very low incidences are similar to our results on PE, although the patients were followed up for different periods of time (1–6 months vs. 90 days).

Others have found incidences of PE after THA in the range 0.1–0.7% with different regimens, with prophylactic treatment from 10–14 days and up to 36 days of rivaroxoban or enoxaparin and up to 6 weeks of aspirin (Mantilla et al. 2002, Lachiewicz and Soileau 2006, 2007, Eriksson et al. 2008, Lassen et al. 2008, Cusick and Beverland 2009). The rate of PE we found is similar to these incidences, although our patients were treated with enoxaparin for only a few days. The standardization in the use of regional analgesia and very early mobilization combined with short hospitalization may be important in achieving such low incidences of PE (Rodgers et al. 2000).

DVT can lead to a post-thrombotic syndrome (PTS) with swelling, varicose veins, pain and discomfort, and hyperpigmentation. However, PTS following TKA has been found to have a similar incidence with or without DVT, and with no major sequelae after ultrasonographically proven DVT (McAndrew et al. 2010); nor was there any substantial increase in risk of PTS after asymptomatic proximal or distal DVT following TKA or THA (Lonner et al. 2006). Also, the largest study on the possibility of PTS after THA failed to find any predictors of PTS—including the infrequent occurrence of DVT (Mant et al. 2008).

We found risks of clinically proven and symptomatic DVT of 0.21% (THA) and 1.15% (TKA) during the 5-year period. Again, these figures compare favorably with studies with longer treatment. Thus, 36 days of treatment with rivaroxaban or enoxaparin after THA resulted in 0.3% and 0.5% risk of DVT, respectively (Eriksson et al. 2008), although LOS or mobilization was not accounted for. Treatment with the same chemoprophylaxis for 10–14 days after TKA led to 1.1% and 2.2% risk of symptomatic DVT with a follow-up of 49 days at most (Lassen et al. 2008), combined with a mean LOS of 6.2 and 6.3 days (the type of mobilization was not mentioned). A multicenter study with prolonged treatment with various types of chemoprophylaxis for 36 ± 9.8 days (59% of patients also used compression stockings) found 1.3% and 2.3% risk of clinically symptomatic DVT after THA and TKA, with a follow-up of 90 days (Samama et al. 2007). This study also found that lack of ambulation was predictive of VTE, as did a study on early mobilization where mobilization within 24 h from the operation resulted in a 30-fold reduction in the risk of postoperative DVT (Pearse et al. 2007). The very early ambulation of our patients supported by our pain protocol with LIA (Andersen et al. 2008 a,b,c, 2009) may thus play an important role in reducing VTE.

Another study involving 6,639 THAs and 8,326 TKAs found cumulative incidences of VTE of 1.8% and 2.3% (Warwick et al. 2007). The data from 100 hospitals in 13 countries were pooled, with varying degrees of chemical and mechanical prophylaxis. Median LOS varied from 3 to 11 days; more than one-third of the patients were discharged to rehabilitation facilities, and more than one-third of the patients had general anesthesia, reflecting the different approaches between departments. However, that study found a lack of compliance with the recommendations regarding the duration of prophylaxis, and found that the risk of VTE extended well beyond LOS in hospital with mean occurrence of VTE of 21.5 and 9.7 days after surgery for THA and TKA patients. Although that study had a very different set-up from our standardized fast-track scenario with prophylaxis only during hospitalization, we found much smaller risks of DVT. We believe that the uniform approach with spinal anesthesia and multimodal analgesia facilitating early mobilization may have been responsible for this difference.

We found only 2 cases of DVT after day 30 (and within 90 days), which coincides with the finding that on average symptomatic DVT occurs 27 days and 17 days after THA and TKA (Dahl et al. 2000). Also, we found 1 of 2 cases of DVT in the proximal veins (proximal to the knee joint) in THA and 3 of 9 cases of DVT in TKA, which corresponds with the findings of Dahl et al.

None of the patients developed PE from DVT within 90 days, which is in accordance with the 2 studies by Kim et al. (2002, 2003) in which proximal thrombi after THA, TKA, and BSTKA were not found to be associated with pulmonary embolism, even without treatment. Dahl et al. (2003) also found only 6 of 50 patients with confirmed PE to have DVT. This may depend on genetic and prothrombotic factors (Kim and Kim 2007).

It should be emphasized that only clinically relevant DVT and PE requiring re-admission were included in our clinical study, as no routine ultrasound examinations were performed. This would lead to an underestimation of the total number of DVTs, as the clinically silent ones are not included. However, quite a few patients were re-admitted suspected of having DVT by their general practitioner. For every one with a positive ultrasound, 8 were negative, as we found only 11 DVTs during the 5 years. As all deaths are accounted for—and as PTS is of little clinical relevance—we have found that clinically symptomatic DVT is a relevant outcome parameter.

We encountered no VTE-related deaths, no PEs, and no DVTs in our study population of 123 patients operated with 246 BSTKAs. The data on bilateral simultaneous total knee arthroplasty suggest that it is safe and has few complications, low mortality, and few VTEs in a fast-track setting with careful selection of the patients (excluding every form of ischemic heart disease) (Kim et al. 2009).

It is both interesting and reassuring from our point of view that we have had no deaths due to VTEs during the last few years in this consecutive, unselected population, and that there is no inverse correlation between risk of VTEs and LOS, where the continuous reduction in LOS during the study period was associated with a shorter duration of prophylaxis.

Our study has several strengths: (1) there was no selection bias since the patients were included in a continuous way based upon referral by the general practioners in the uptake area of the hospital; (2) The patients operated at our hospital are representative of THA and TKA patients; age, sex, co-morbidities etc. are typical of all patients and have been described in detail (Husted et al. 2008); (3) there was 100% follow-up; and (4) the results of our study can be expected to be the same in other departments, as our patients are typical unselected arthroplasty patients—provided that our fast-track protocol is copied. However, in our study only historical comparisons are given, as there have been no randomized controlled trials (RCTs) on fast-track surgery with early mobilization compared to a more conventional approach without early mobilization, despite the fact that early mobilization appears to be associated with lower rates of DVT (Pearse et al. 2007, Xing et al. 2008).

In conclusion, our fast-track THA and TKA regimen with early mobilization and short hospitalization—and subsequently a very short duration of LMWH chemoprophylaxis—resulted in very low risks of clotting-related death, PE, and DVT. This study was not designed (i.e. regarding the set-up and statistical power) to identify these parameters as the sole determinants of low risk of VTE, as the underlying reasons are indeed multifactorial. Rodgers et al. (2000), Pearse et al. (2007), and Xing et al. (2008) have suggested that earlier mobilization has an important role in reduction of VTE and our study appears to confirm this. A large, properly designed RCT with only the timing (and therefore the amount) of mobilization after surgery varying between groups is needed to determine the true importance of mobilization (but it would require several thousand patients).

As the risks of VTE in the present study were lower or comparable to the reported incidences in the literature from studies based on the recommended duration of prophylaxis of at least 10 days for both THA and TKA, or up to 35 days for THA, we suggest that the principles of chemical thromboprophylaxis should be reconsidered, taking into account the clinical set-up, the time of first mobilization, and the length of hospital stay.
